# A systematic review and meta‐analysis of the association between mood disorders and Alzheimer’s dementia

**DOI:** 10.1002/alz.084635

**Published:** 2025-01-09

**Authors:** Liwei Ma, Benjamin Goudey, Colin L. Masters, Liang Jin, Yijun Pan

**Affiliations:** ^1^ Florey Institute of Neuroscience and Mental Health, Parkville, VIC Australia; ^2^ University of Melbourne, Parkville, VIC Australia; ^3^ Florey Institute of Neuroscience and Mental Health, University of Melbourne, Parkville, VIC Australia; ^4^ ARC Training Centre in Cognitive Computing for Medical Technologies, Parkville, VIC Australia; ^5^ Monash University, Parkville, VIC Australia; ^6^ Tohoku University, Sendai Japan

## Abstract

**Background:**

Anxiety and depression are mood disorders that can manifest decades before the onset of dementia, including mild cognitive impairment (MCI) and Alzheimer’s disease (AD). A systematic review with meta‐analysis was conducted to investigate whether anxiety/depression modifies the risk of MCI/AD.

**Method:**

Embase, Global Health, MEDLINE, APA PsycInfo (from inception to July 2023) were systematically searched for well‐structured cohort studies examining the association between anxiety/depression and dementia. Meta‐analyses were performed, and summary relative risks (RRs with 95% confidence intervals [95% CI]) were calculated using a random effects model. The quality of the evidence was assessed using the GRADE tool.

**Result:**

Thirty‐nine studies met the inclusion criteria. Anxiety was significantly associated with MCI (six studies, RR 1.50 [1.11‐2.03], p = 0.018) but not with AD (ten studies, RR 1.31 [0.94‐1.81], p = 0.099). Depression was significantly associated with both MCI (fourteen studies, RR 1.35 [1.08‐1.68], p = 0.012) and AD (nineteen studies, RR 1.57 [1.26‐1.96], p < 0.001).

**Conclusion:**

The meta‐analysis has demonstrated that anxiety is associated with increased incidence of MCI, and depression is associated with increased incidence of both AD and MCI. The current study provides epidemiological evidence that anxiety and depression have potential additive effects to the clinical evolution of MCI/AD.

